# Conservation Action Based on Threatened Species Capture Taxonomic and Phylogenetic Richness in Breeding and Wintering Populations of Central Asian Birds

**DOI:** 10.1371/journal.pone.0110511

**Published:** 2014-10-22

**Authors:** Manuel Schweizer, Raffael Ayé, Roman Kashkarov, Tobias Roth

**Affiliations:** 1 Naturhistorisches Museum der Burgergemeinde Bern, Bern, Switzerland; 2 SVS/BirdLife Switzerland, Zurich, Switzerland; 3 National University of Uzbekistan, Tashkent, Uzbekistan; 4 Uzbekistan Society for the Protection of Birds, Tashkent, Uzbekistan; 5 University of Basel, Zoological Institute, Basel, Switzerland; 6 Hintermann & Weber AG, Reinach, Switzerland; Institute of Biochemistry and Biology, Germany

## Abstract

Although phylogenetic diversity has been suggested to be relevant from a conservation point of view, its role is still limited in applied nature conservation. Recently, the practice of investing conservation resources based on threatened species was identified as a reason for the slow integration of phylogenetic diversity in nature conservation planning. One of the main arguments is based on the observation that threatened species are not evenly distributed over the phylogenetic tree. However this argument seems to dismiss the fact that conservation action is a spatially explicit process, and even if threatened species are not evenly distributed over the phylogenetic tree, the occurrence of threatened species could still indicate areas with above average phylogenetic diversity and consequently could protect phylogenetic diversity. Here we aim to study the selection of important bird areas in Central Asia, which were nominated largely based on the presence of threatened bird species. We show that although threatened species occurring in Central Asia do not capture phylogenetically more distinct species than expected by chance, the current spatially explicit conservation approach of selecting important bird areas covers above average taxonomic and phylogenetic diversity of breeding and wintering birds. We conclude that the spatially explicit processes of conservation actions need to be considered in the current discussion of whether new prioritization methods are needed to complement conservation action based on threatened species.

## Introduction

The IUCN Red List of threatened species (henceforth IUCN RL) constitutes a de facto baseline reference for many conservation decisions [1], [2]. The importance of the RL as a conservation tool derives not only from the standardized methodology for assessing the threat levels of each species, but also from the wealth of accessible data, collected to support these assessments [3]. Data from the IUCN RL are being used as basis to guide management of natural resources at multiple scales, including individual sites, national scales such as national biodiversity strategies and action plans, or multi-national agreements such as the convention on international trade in endangered species [2]. Taken together, species listed in the IUCN RL (henceforth RL species) and local Red Lists currently play a major role across the world when allocating conservation resources [4].

Apart from threat status, the evolutionary history of species was suggested as an additional currency to allocate conservation resources [5], [6]. Phylogenetic diversity (PD hereafter) is a biodiversity measure based on the evolutionary history (i.e., phylogenetic relationships) between taxa [7]. Here, we use PD as a generic term to refer to any of the metrics in the jungle of different indices based on evolutionary relationships between species [8], [9]. PD can be used as a measure for evolutionary processes [8] and as a proxy for ecosystem functioning and stability as more phylogenetically diverse assemblages potentially maintain higher function [9]–[12]. Hence, safeguarding PD would optimize the preservation of evolutionary potential and possibly ecosystem functioning, and is thus relevant from a conservation point of view.

To date, information on evolutionary relationships among species has not been widely integrated into conservation planning [8], [9], and the IUCN RL, which is by far the largest and most comprehensive assessment of conservation status, has been identified as acerbating the problem [13]. The main critic is, that the IUCN RL was designed to indicate the risk of species extinction without indicating the consequences of losing a given species [14]. It has been argued that while loosing a random species could have only minor consequences for ecosystem stability or functioning, loosing an evolutionary unique species might reduce the potential of communities to react to environmental change as a consequence of reduced genetic diversity or evolutionary potential in general [15]. This argument has been put forward to the notion that conservationists need to evaluate whether the IUCN RL categories represent species with diverse evolutionary histories to decide whether relying on RL species alone can help to conserve communities with diverse evolutionary histories [14], [16]. Based on the assumption that RL species usually do not capture greater PD than expected by chance (i.e., their PD does not significantly differ from the PD of randomly selected species), the practice of investing conservation resources based on RL species was challenged as this would be the same as protecting species at random [14].

However, these statements seem to neglect the fact that conservation action is a spatially explicit process [17]. Threatened species are usually protected by protecting their sites of occurrence and/or by measures to conserve their habitats. Recognition and safeguarding of sites with threatened species has been a well-accepted and successful conservation strategy to face the human caused biodiversity crisis [18]. RL species have high extinction risks because they are sensitive to human-induced change and/or because they have very specific habitat requirements (e.g. [19], but see [20]). Areas where RL species occur are likely areas comprising distinctive and often underrepresented habitats, and thus, conservation efforts to protect RL species have also helped to avoid the deterioration in status of least concern species in many cases e.g. [19], [21], [22], [23]. Therefore, site-based conservation approaches may preserve overall species diversity by focusing on conserving viable populations of RL species in their natural habitats [24]. Consequently, even if RL species are usually not evenly distributed over the phylogenetic tree [14], their occurrence patterns may correlate with areas of above average species richness and/or areas of higher PD.

We here aim at testing this hypothesis by studying the recent selection of 267 important bird areas (IBA) in Central Asia. We particularly wanted to elucidate whether the current IBA approach does or does not automatically cover the conservation of PD of all species breeding in the region (i.e. breeding birds) and all species using Central Asia as wintering grounds mainly from December to February (i.e. wintering birds). We analysed breeding and wintering birds separately because breeding and wintering bird communities often differ in temperate regions such as Central Asia [25]. Moreover, since bird populations are also limited by factors affecting survival and physical conditions during non-breeding seasons [26], conservation measures should be effective in protecting breeding as well as non-breeding populations of focal species. The IBA network initiated by BirdLife International already in the 1980s [27] is a widely recognized approach to designate comprehensive networks of sites to protect bird populations of conservation importance [28]. IBAs are selected nationally using objective and quantitative criteria based chiefly on the presence of birds of global conservation concern and assemblages of biome-restricted bird species (http://www.birdlife.org/). We here show that the RL species occurring in Central Asia indeed do not capture greater PD than expected by chance from the phylogenetic tree of the birds that regularly breed or winter in Central Asia. Based on this, we tested whether the 267 IBAs of Central Asia covered on average and in total more species, more RL species and a higher PD using two different metrics [8], [29], [30] than random selections of 267 sites. We moreover provide a graphical illustration for the spatial distribution of the hotspots for the different indices to identify areas of high biodiversity concern.

Analyses of spatial patterns of species ranges and the evolutionary history of regional species assemblages are rare because of the shortage of distributional information and phylogenetic data [17] and we are not aware of a single study that compared breeding and wintering species assemblages. Here, we used a phylogenetic hypothesis on all Central Asian breeding and wintering birds from Jetz et al. [31] and analysed the distribution maps from a recently published field guide [25]. To our knowledge, the results of our study provide for the first time evidence that a site-based conservation approach mainly focusing on threatened species as a side effect also covers the conservation of phylogenetic diversity not only for breeding but also for wintering populations.

## Materials and Methods

### Study area and important bird area IBA

We used Central Asia as study area comprising the countries Afghanistan, Kazakhstan, Kyrgyzstan, Tajikistan, Turkmenistan and Uzbekistan. This area is characterised by high mountain ranges including the Altai, the Tien Shan, the Pamir, the Hindu Kush or the Kopet Dag, and extensive, mostly semi arid plains. It contains a variety of different biomes ranging from Saharo-Sindian and Eurasian semi-deserts and deserts, Eurasian steppes, Irano-Turanian mountains to the Eurasian high montane region as well as from Sino-Himalayan subtropical and temperate to boreal forests [25], [32]–[34].

**Figure 1:**
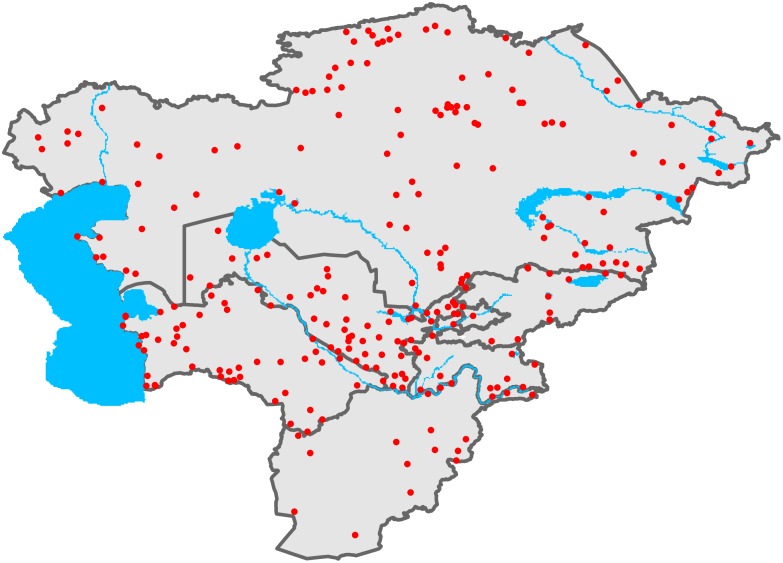
Study area. Points indicating the locations of the 267 important bird areas (IBAs) in Central Asia (comprising the countries Afghanistan, Kazakhstan, Kyrgyzstan, Tajikistan, Turkmenistan and Uzbekistan).

The information on IBAs from Central Asia was downloaded on 04.11.2013 from http://www.birdlife.org/datazone/site/search with the search-term “Central Asia”. From the search result we excluded all sites with the term “Russia (Central Asian)” in the “country” column. The search resulted in a total of 267 IBAs with 16 IBAs in Afghanistan, 121 IBAs in Kazakhstan, 11 IBAs in Kyrgyzstan, 18 IBAs in Tajikistan, 50 IBAs in Turkmenistan and 51 IBAs in Uzbekistan (Fig. 1). The average area of these IBAs is 96′887 ha.

### Ethics statement

Since we analysed data from a recently published field guide, the BirdLife database and a recently published phylogenetic hypothesis, no fieldwork was conducted and no specific permits were required for the described study.

### Distributional data and phylogeny

The distributions of 507 bird species that are regularly breeding or wintering in Central Asia were obtained from the distribution maps published in a recent field guide to the birds of Central Asia [25]. The distribution maps were based on information from regional handbooks and monographs on the different species groups, locality data from specimens of the collections of the National University of Uzbekistan and the University of Samarkand, as well as on field observation data from various sources [25]. We obtained all distribution maps as single files in tagged image file format (tiff) with a resolution of 0.03°×0.03° resulting in 513,631 grid cells covering the entire study area.

We used the phylogeny on all birds regularly breeding or wintering in Central Asia as provided by Jetz et al. [31]. Since some recent splits of mostly non-sympatric sister-species were not contained in Jetz et al. [31], we lumped the distribution information of the following species: *Carpodacus grandis/rhodochlamys, Corvus corone/cornix, Falco cherrug/altaicus, Lanius excubitor/lahtora, Lanius isabellinus/phoenicuroides, Melanitta fusca/deglandi, Nucifraga caryocatactes/multipunctata, Oriolus oriolus/kundoo, Parus major/cinereus, Parus montanus/songarus, Passer domesticus/indicus, Pyrrhula pyrrhula/cineracea, Riparia riparia/diluta, Sylvia minula/curruca*. Thus, we analysed the distribution information of 490 species or taxonomic groups in total. To obtain a phylogenetic hypothesis, we first pruned the phylogenetic tree provided by Jetz et al. [31] to contain those 490 species and sampled 1000 trees from the pseudo-posterior distribution that was based on the Hackett backbone [31]. From these 1000 trees, we then calculated a maximum clade credibility tree using mean node heights with the software TreeAnnotator v. 1.7.5 of the BEAST package [35]. The resulting tree was used for all further analyses on phylogenetic diversity (Fig. S1).

### Statistical analyses

We computed the total branch length of the phylogenetic tree that connected all Central Asian RL species according to BirdLife International and IUCN (i.e., near threatened, vulnerable, endangered, critically endangered, and data deficient [3]). We included *Acrocephalus orinus*, which was the only data deficient species in our sample because it is likely a threatened species based on its small breeding range and the intense pressures acting on the species’ habitat [36]. We then used a Null model approach to infer whether the total branch length of the RL species differed significantly from what would be expected by chance. We generated 1000 random trees by shuffling the taxon labels across the tips of the phylogenetic tree of all species from Central Asia [14]. We used the 2.5% and 97.5% percentiles of the total branch length calculated for the RL species from the 1,000 random trees as an estimate of the confidence interval under the Null hypothesis that the RL species are randomly distributed over the phylogenetic tree of Central Asian birds. A value of the estimated total branch length of RL species outside the confidence interval would suggest that RL species are not randomly distributed over the phylogenetic tree.

We geo-referenced the distribution maps using the software Quantum-GIS [37]. For every grid cell and every species, we compiled the information whether a species was breeding and/or wintering. Using this information, four different measures of bird diversity for each grid cell of the study area were calculated for breeding and wintering bird species separately: 1) the total number of species (i.e. taxonomic richness), 2) the number of RL species, 3) phylogenetic richness calculated as the sum of branch lengths between root and tips for the species in a community [8], [29], 4) phylogenetic distinctiveness calculated as the sum of all branch lengths connecting two species averaged across all species in a community [8], [30]. We used phylogenetic richness and phylogenetic distinctiveness as two distinct measures of PD because the conservation implication of the first measure is straightforward but it is strongly linked to taxonomic richness, while the second is independent of taxonomic richness but it will increase if closely related species go extinct [8].

To test whether the selection of IBAs was effective in including bird diversity according to the four above measures of diversity, we compared the 267 grid cells that contained the centre of an IBA (i.e., the IBA grid cells) with the same number of randomly selected grid cells (i.e., the random grid cells). The random grid cells were drawn from all 513,631 grid cells with each cell having equal inclusion probability. The random selection of grid cells was repeated 1,000 times. For each set of random grid cells we then calculated IBA effectiveness as follows.
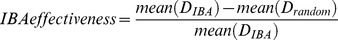
Where 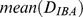 refers to the average of one of the four bird diversity measures (see above) of the IBA grid cells and 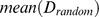 to the average of the same bird diversity measure of the random grid cells. Thus, for example, if the average taxonomic richness of IBAs was larger than the average taxonomic richness of randomly selected sites, we would conclude that IBAs were effective in conserving the taxonomic richness (i.e. 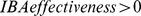), because the average taxonomic richness of an IBA according to the distribution maps was higher than expected by chance. However, protecting sites based on high average diversity may dismiss specialised species that live in areas with generally low diversity (e.g. species living in high alpine habitats with comparatively low taxonomic richness). Therefore, we additionally applied a complementary approach [38] and thus asked whether each of the four diversity measures for all IBAs in total was higher compared to the randomly selected grid cells. Thus, for all species occurring in at least one IBA grid cell, we calculated each of the four diversity measures and compared them with the corresponding diversity measure of all species occurring in at least one of the randomly selected grid cells.

To allow a better understanding of why the effectiveness of IBAs differed between the measures of diversity, we produced maps for the distribution of the 10% of grid cells with the highest taxonomic richness, the highest number of red list species, the highest phylogenetic richness and the highest phylogenetic distinctiveness inferred for both breeding and wintering distributions. All analyses were performed using the software R version 3.0.2 [39]. To extract the information on the species' distribution from the tiff-files we used the R package raster [40]. The calculation of the phylogenetic richness and the phylogenetic distinctiveness was done with the functions *pd* and *mpd*, respectively,from the R-package picante [41].

## Results

The total branch length of the 27 RL species regularly wintering or breeding in Central Asia was 1260.6. As this value was well within the confidence interval under the Null hypothesis that the RL species are randomly distributed over the phylogenetic tree of Central Asian birds (1104.7–1484.3), there is no indication that RL species capture less or more phylogenetic diversity than expected by chance.

According to the distribution maps, the average (mean ± SD) taxonomic richness in the 267 grid cells that contained the centres of the IBAs was 104.1±36.9 breeding bird species and 58.8±30.6 wintering species. In total, 457 species occurred in at least one of the IBA grid cells during summer and 329 species in at least one of the IBA grid cells during winter, which corresponded to exactly the same percentage of 97.9%,for both breeding and wintering species. The species that occurred in at least one of the IBA grid cells during summer made up 98.9% of the total branch length of the phylogenetic tree containing all species breeding in Central Asia. For wintering birds, the species occurring in at least one of the IBA grid cells made up 97.5% of the total branch length of the phylogenetic tree of all wintering birds. For the RL species, the average taxonomic richness in IBAs was 5.7±2.6 for breeding species and 1.9±2.0 for wintering species. In total, 25 RL species occurred in at least one of the IBA grid cells during summer and 14 RL species during winter, which corresponded to 96.2% and 100% of all RL species for breeding and wintering birds, respectively.

**Figure 2:**
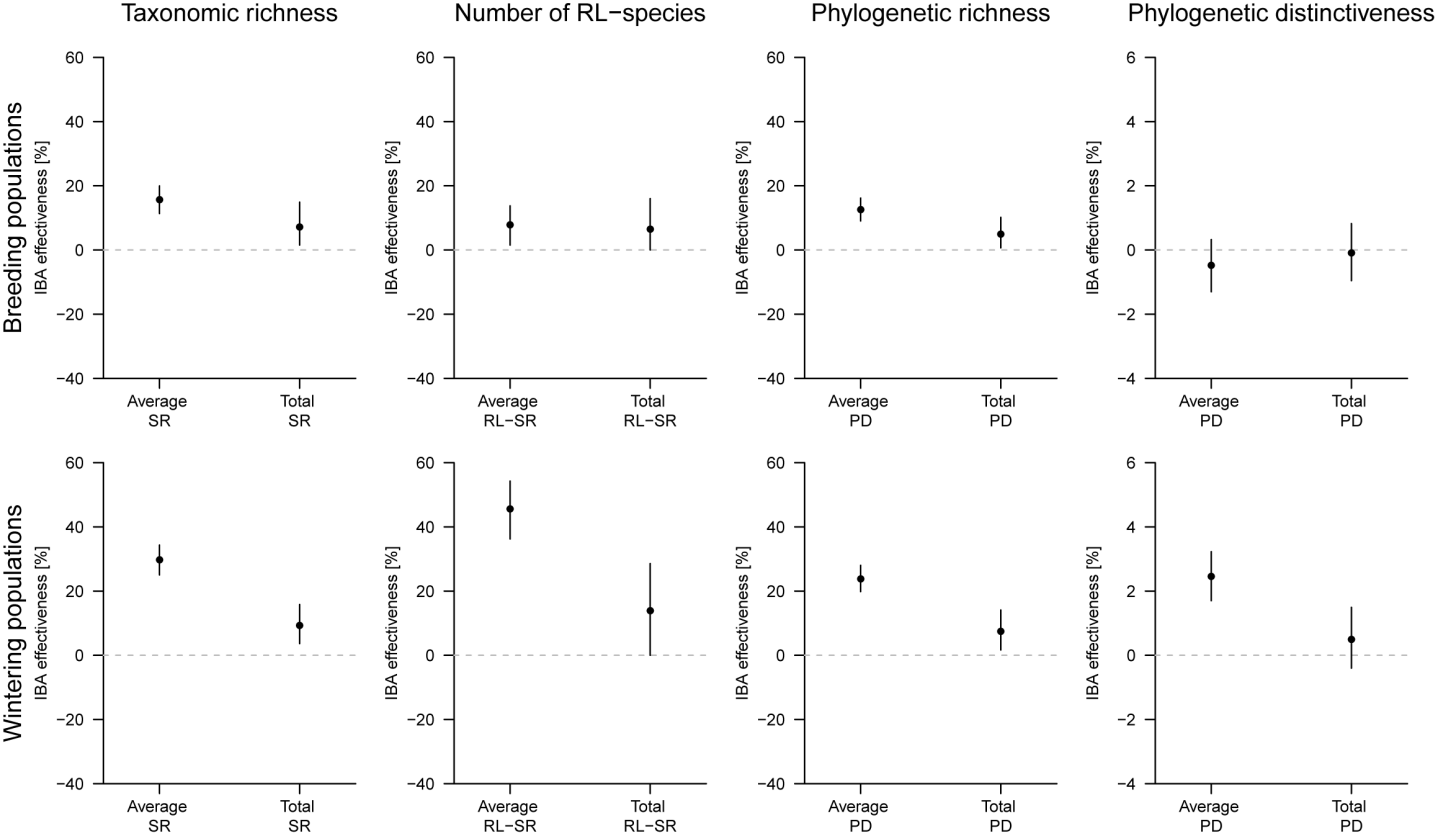
Effectiveness of important bird areas (IBA) to include bird diversity. The effectiveness of IBAs for Central Asia was compared with the same number of randomly selected grid cells and is given for the number of species (taxonomic richness, first column), the number of red list (RL) species (second column), the phylogenetic richness (total branch length; third column) and the phylogenetic distinctiveness (mean pairwise distance of branch length of all species pairs, fourth column) inferred from the breeding distributions (upper row) and wintering distributions (lower row) of regularly occurring birds in Central Asia. Effectiveness was measured either as the average diversity measures of all IBAs (‘Average’) or as the measure of all species occurring at least in one IBA (‘Total’) and expressed as the IBA measure minus the measure from the random sample divided by the IBA measure. Points give the average and lines give the 2.5% and 97.5% quantiles of the 1,000 simulations. SR = Species richness.

The effectiveness of IBAs in representing diversity was measured either as the average of the respective diversity measure across all IBAs (‘Average’) or as the measure of all species occurring in at least one IBA in a complementary approach (‘Total’) and compared to the random selection of sites. Irrespective of whether we used the ‘average’ or the ‘total’ approach to measure effectiveness, and irrespective of whether we considered breeding or wintering birds, the taxonomic richness, the number of RL species and the phylogenetic richness of birds were higher in IBAs compared to randomly selected sites. The same was true for the phylogenetic distinctiveness of wintering birds considering the ‘average’ of all IBAs. In contrast, the phylogenetic distinctiveness of breeding birds according to both approaches and the ‘total’ phylogenetic distinctiveness of wintering species did not significantly differ between IBAs and randomly selected sites (Fig. 2).

**Figure 3:**
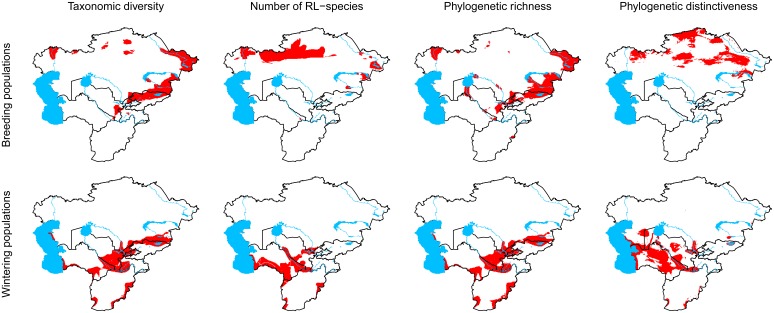
Distribution of bird diversity hotspots in Central Asia. The red area in the figures gives the 10% of grid cells with the highest number of species (taxonomic richness, first column), the highest number of red list species (second column), the highest phylogenetic richness (total branch length; third column) and the highest phylogenetic distinctiveness (mean pairwise distance of branch length of all species pairs, fourth column) inferred from the breeding distributions (upper row) and wintering distributions (lower row) of regularly occurring birds in Central Asia.

The spatial distribution of the 10% grid cells with the highest taxonomic richness over Central Asia was similar to the distribution of the 10% grid cells with the highest phylogenetic richness for both, breeding and wintering populations (Fig. 3). In contrast, the distribution of the 10% sites with the highest number of RL species resembled that of the 10% sites with the highest phylogenetic distinctiveness. The 10% grid cells with the highest numbers of the four diversity measures were found to be located much more to the south for wintering populations compared to breeding populations (Fig. 3.). This may simply reflect the trend that wintering areas of non-resident species are located further south than breeding areas.

## Discussion

Although phylogenetic diversity might be relevant from a conservation point of view [5], [9], its limited role in practical nature conservation has fostered an academic discussion about why phylogenetic diversity is so little used in nature conservation [8], [13], [14], [42], [43]. One of the main arguments suggests that the practice of investing conservation resources based on the IUCN RL, is a major reason for the slow integration of phylogenetic diversity in nature conservation planning [13], [14].

While the IBA strategy clearly focuses on the protection of rare and endangered as well as range restricted species [27], [28], the evolutionary uniqueness of given species is currently not considered as an argument to designate IBAs. We showed that although threatened species occurring in Central Asia do not capture greater phylogenetic diversity from the phylogenetic tree of Central Asian birds than expected by chance, the selection of IBAs nonetheless covered above average taxonomic richness and phylogenetic richness of breeding and wintering birds. These results suggest that even though RL species were randomly distributed over the phylogenetic tree, they will effectively contribute to the protection of the tree of life when their habitats and sites of occurrence are protected. For example, the globally threatened Sociable Lapwing needs heavily grazed swards for breeding, and these are also the areas, where a number of other species reach their highest densities [44]. Similarly, the riverine woodlands identified as the breeding habitat of the Large-billed Reed Warbler host a considerable number of least concern species including relatively scarce and/or biome-restricted species [36]. Outside our study region, there are numerous examples of conservation action targeting species figuring on the IUCN RL or a national RL and benefiting less or not threatened species, including focus areas managed for Little Bustard *Tetrax tetrax* or Grey Partridge *Perdrix perdrix* benefiting Skylark *Alauda arvensis* and Corn Bunting *Emberiza calandra* or Wryneck *Jynx torquilla* benefiting from nestboxes installed for Hoopoe *Upupa epops* [21], [45], [46]. The protection of RL species is thus likely to benefit non-threatened species, too. Hence, even though the IBA strategy does not consider the functional role of species, its implementation in Central Asia does indirectly protect phylogenetic diversity and thus ecosystem functionality, which is potentially linked to the latter. We thus conclude that recognizing conservation being a spatially explicit process is important when deciding how to integrate evolutionary processes into conservation planning [17].

Although threat categories do not cover more phylogenetic and functional diversity in birds of Brazil as has been shown recently [14], protected areas in this country selected based on the occurrence of RL species may nonetheless indirectly safeguard evolutionary and functionally unique species as in Central Asia. However, it has to be stressed that the results from our study cannot be generalized, and it has to be tested for different regions separately whether spatially explicit conservation practices based chiefly on threatened species like the IBA approach also help to conserve phylogenetic diversity. For breeding birds in France for example, a rather poor geographical match between different diversity components was found [47].

Unfortunately, however, analyses of spatial patterns of species ranges and the evolutionary history of regional species assemblages are often hindered by the lack of distributional information and phylogenetic data [17]. In our study, we used the maps from a recently published field guide [25], which provided up-to-date distribution information for all species occurring in the region. We acknowledge that maps are only approximations of true distributions of species and do not necessarily indicate naturally occurring communities. Nonetheless, we think that using the information from distribution maps of field guides with appropriate resolution as we did in our study can provide important data sources to answer contemporary questions in applied ecology and conservation biology [48].

Effective protection measures have to take into account intra-annual variation in the geographical distribution of focal taxa and accordingly the designation of IBAs is not restricted to breeding ranges, but also includes the non-breeding areas of threatened species as well as concentrations of individuals of any species. Surprisingly however, there seems to be no study considering seasonal variation of phylogenetic diversity in communities. In our study we found that during summer the phylogenetic distinctiveness of IBAs did not differ from randomly selected sites, while during winter the average phylogenetic distinctiveness was higher in IBAs compared to randomly selected sites. We believe that our study is the first to show that analyses of spatial patterns of species ranges and the evolutionary history of regional species assemblages may differ between breeding and wintering population. Hence, the protection of phylogenetic diversity needs to account for intra-annual variation in species distributions.

The spatial distribution of the hotspots for taxonomic richness over Central Asia resembled the distribution of hotspots for phylogenetic richness, while the distribution of hotspots for RL species was more similar to the distribution of hotspots for phylogenetic distinctiveness. These results fit rather well with other studies showing that phylogenetic richness is mathematically linked to taxonomic richness [49] and showing that phylogenetic distinctiveness is often correlated with rarity [16], [50]. Mountainous regions such as the Altai, Hissor-Alay, Jungarian Alatau and the Tien Shan [25] have been found to be areas of high taxonomic and phylogenetic richness especially for breeding species. This fits in the general pattern with mountains often being biodiversity hotspots and several Central Asian mountains having been identified as areas of high global conservation priority [18]. Our results also add to the discussion of doing conservation by proxy [51] by suggesting that taxonomic richness could be used as an indicator for areas with high phylogenetic richness and number of RL species might be used as an indicator for areas with high phylogenetic distinctiveness. However, the contrasting results of the application of phylogenetic richness and phylogenetic distinctiveness in our study underpin that we need a solid conceptual basis and reliable guidance in the jungle of the different indices before they can be used for conservation assessments [8]. As mentioned above, phylogenetic distinctiveness can be maximised by removing all but one of any group of closely related species. Hence this index has to be used with caution and must be considered as a questionable indicator for site-based conservation action.

## Conclusion

Even though the current bird conservation strategy in Central Asia has a clear focus on threatened species, which are randomly distributed over the phylogenetic tree, current conservation action seems to capture a high proportion of taxonomic and phylogenetic diversity. These results suggest that the on-going discussion of whether new prioritization methods are needed to complement conservation action based on threatened species should explicitly consider the spatially explicit nature of conservation actions. However, a spatial and intra-annual congruence between different biodiversity components cannot a priori be expected and needs to be tested case specifically.

## Supporting Information

Figure S1 Maximum clade credibility tree using mean node heights of all birds regularly breeding or wintering in Central Asia.
(PDF)
