# Supplementary material for: Conservation Action Based on Threatened Species Capture Taxonomic and Phylogenetic Richness in Breeding and Wintering Populations of Central Asian Birds
Source: PLoS One. 2014 Oct 22;9(10):e110511. doi: 10.1371/journal.pone.0110511 (PMC4206344; doi:10.1371/journal.pone.0110511)
Supplement: Figure S1 — Maximum clade credibility tree using mean node heights of all birds regularly breeding or wintering in Central Asia. (PDF) [file pone.0110511.s001.pdf]

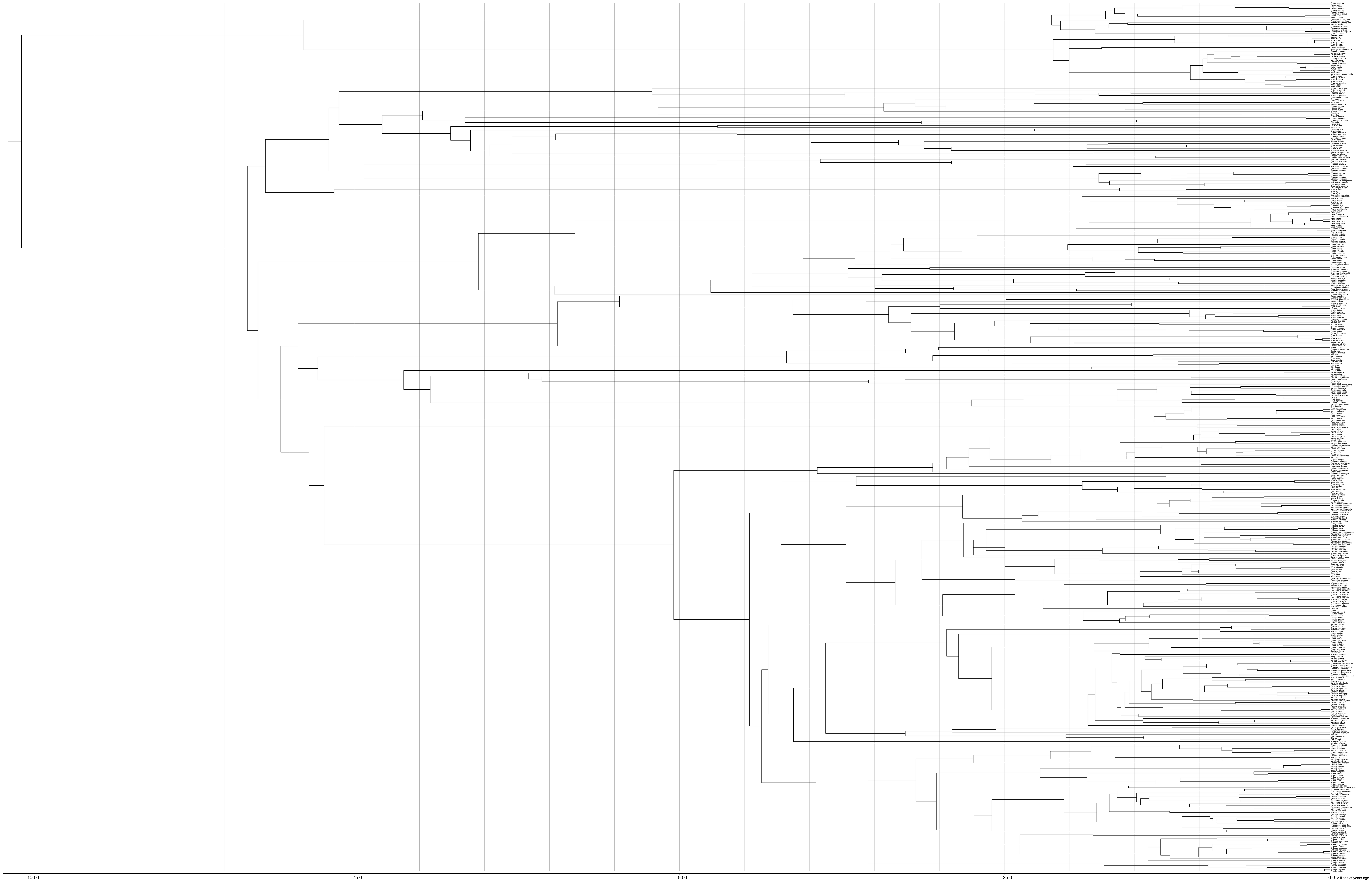
100.0
75.0
50.0
25.0
0.0 Millions of years ago
